# Prior exposure to capture heightens the corticosterone and behavioural responses of little penguins (*Eudyptula minor*) to acute stress

**DOI:** 10.1093/conphys/cov061

**Published:** 2016-01-19

**Authors:** Gemma Carroll, Emma Turner, Peter Dann, Rob Harcourt

**Affiliations:** 1 Department of Biological Sciences, Faculty of Science and Engineering, Macquarie University, Sydney, NSW 2109, Australia; 2 Research Department, Phillip Island Nature Parks, PO Box 97, Cowes, Phillip Island, VIC 3922, Australia

**Keywords:** Endocrinology, habituation, human disturbance, monitoring, seabird, tourism

## Abstract

Studies of physiology can provide important insight into how animals are coping with challenges in their environment and can signal the potential effects of exposure to human activity in both the short and long term. In this study, we measured the physiological and behavioural response of little penguins (*Eudyptula minor*) that were naïve to human activity over 30 min of capture and handling. We assessed relationships between corticosterone secretion, behaviour, sex and time of day in order to characterize the determinants of the natural stress response. We then compared the response of these naïve penguins with the responses of female little penguins that had been exposed to research activity (bimonthly nest check and weighing) and to both research activity (monthly nest check and weighing) and evening viewing by tourists. We found that corticosterone concentrations increased significantly over 30 min of capture, with naïve penguins demonstrating a more acute stress response during the day than at night. Penguins that had previously been exposed to handling at the research and research/visitor sites showed elevated corticosterone concentrations and consistently more aggressive behaviour after 30 min compared with naïve birds, although there were no significant differences in baseline corticosterone concentrations. Our findings demonstrate that these little penguins have not habituated to routine capture, but rather mount a heightened physiological and behavioural response to handling by humans. Less invasive research monitoring techniques, such as individual identification with PIT tags and automatic recording and weighing, and a reduction in handling during the day should be considered to mitigate some of the potentially negative effects of disturbance. Given the paucity of data on the long-term consequences of heightened stress on animal physiology, our study highlights the need for further investigation of the relationship between the corticosterone stress response and fitness outcomes, such as breeding success and survival.

## Abstract (precis)

The hormonal stress response was determined for little penguins naïve to human activity and little penguins exposed to research and tourism. Penguins exposed to human activity showed elevated stress hormone levels 30 minutes after capture, indicating that they may be sensitised rather than habituated to some types of human interaction.

## Introduction

Research and tourism programmes centred on the observation and monitoring of animals are becoming increasingly widespread. These activities may contribute to conservation potential through increased ecological knowledge about animal populations (Martin *et al*., 2007), public awareness (Ballantyne *et al*., 2011) and financial gain for local organizations and communities (Wilson and Tisdell, 2003). However, the cost of these activities to animals at both the individual and the population level is poorly understood, and human–wildlife interactions often entail trade-offs between the welfare of individual animals and potential conservation benefits for the wider population (McMahon *et al*., 2012). By understanding the nature and magnitude of the stress imposed on animals by different types of interactions with humans, there is an opportunity to make simple and immediate modifications to research and tourism protocols to minimize disruption and maximize the positive outcomes for conservation and sustainable ecotourism.

The way that animals experience anthropogenic disturbance has been assessed using a variety of methods, each with benefits and drawbacks (see Gill, 2007; Tarlow and Blumstein, 2007). Long-term consequences of disturbance can be identified at the population level using changes in demographic parameters (French *et al*., 2011). In the short term, behavioural responses such as flight initiation have often been used as measures of disturbance (Weston *et al*., 2012). However, this can be problematic particularly when evolution has favoured strategies such as silence and motionlessness for predator evasion or when an animal is committed to defending a nest during breeding (Ellenberg *et al*., 2013). In these cases, physiological indicators of stress may provide the most appropriate means of quantifying the way that animals experience acute disturbance by human activity. Physiological traits may also provide insights into the way that stress may affect the overall fitness of animals and how this may differ with individual experience or between sexes or life-history stages (Wingfield, 2013). Glucocorticoids provide a physiological parameter for indicating the character and magnitude of stress in many vertebrate species. During a stress response, temporarily high concentrations of glucocorticoids are secreted by the hypothalamic–pituitary–adrenal axis, which confer short-term benefits to an individual and enable it to cope effectively with unexpected stimuli in its environment (Sapolsky *et al*., 2000). Benefits include the mobilization of energy stores and glucose sparing for the brain. If glucocorticoid concentrations are not able to return to baseline quickly because of an animal’s inability to habituate to a stress-eliciting stimulus, a state of chronic stress may be entered, in which energy is diverted that would ordinarily be allocated for activities such as growth, reproduction, foraging and homeostasis. This can contribute to an energy deficit that results in decreased body condition or ‘allostatic overload’ (McEwen and Wingfield, 2003).

The little penguin (*Eudyptula minor*) is the only penguin native to mainland Australia and has a significant tourism industry built around viewing it in the wild (Dann and Chambers, 2013). Little penguins have also been the subjects of extensive research efforts, with a large body of literature describing their ecology, behaviour and physiology (e.g. Saraux *et al*., 2011; Mouterde *et al*., 2012; Dann and Chambers, 2013; Pelletier *et al*., 2014). Although some well-managed little penguin populations have increased significantly in recent decades (Dann, 2013; Sutherland and Dann, 2014), other populations are in decline, and it is important to ensure that research and tourism-related activities are not potential threats (e.g. Dann *et al*., 2014). Measured responses of little penguins to human activity vary depending on the type and intensity of the interaction, from reductions in breeding success caused by the presence of holidaymakers at a breeding colony (Klomp *et al*., 1991) to no indications of adverse effects caused by regulated observation by tourists (Dann, 1992).

The aim of the present study was to assess the acute corticosterone stress response of little penguins on Phillip Island, a reserve that has both a dedicated long-term penguin research programme and the largest Australian tourist operation centred on viewing penguins coming ashore at dusk (∼540 000 visitors per year in 2014–15). The aim of the first part of this study was to assess the natural stress response in penguins that had not been exposed to research or tourism and to determine the effects of sex and time of day on corticosterone concentrations during capture and handling. Second, we explored whether this response differed in penguins that had been exposed to research activities only (bi-monthly checking and weighing) or to a combination of research and tourism (monthly checking and weighing, and evening viewing by visitors as the birds walk up the beach to their nests). In doing this work, we aimed to quantify how penguins respond physiologically to different levels and types of human activity and to provide specific recommendations for tourism and research protocols that might benefit penguin populations and inform other long-term conservation monitoring programmes.

## Methods

### Field site and study groups

The study was conducted from 12 to 20 April 2000 on Phillip Island (38°31′S, 145°08′E), off the coast of Victoria, Australia. Phillip Island lies 70 km southeast of Melbourne and is ∼10 000 ha in area (Reilly and Balmford, 1975). The island is composed of sandy shores backed by dunes, steep, vegetated cliffs or bare, rocky headlands.

In April, adult little penguins on Phillip Island are in their non-breeding season and have recently completed their post-nuptial moult and associated fast. Eighty-nine adult birds were captured during the study period, from three sites on the Summerland Peninsula. For night captures, the site was visited 12 h before the intended capture period to mark unoccupied burrows within a designated capture area. For day captures, the site was visited 24 h before the intended capture period to mark unoccupied burrows within a designated capture area, and penguins were captured the following day to ensure that penguins had not been disturbed by burrow marking.

We captured male and female penguins during the night (22.00–03.00 h) and day (10.00–15.00 h) from a control site, in which penguins nest in natural burrows and had no exposure to human activity prior to this study. We captured only female penguins during the night from the research and research/visitor sites to compare with the female penguins captured at night at the control site. The research site is located 1500 m northeast of the control area, and the penguins in this area nest in wooden nest boxes. Penguins in this area are handled for weighing and identification twice a month by researchers from Phillip Island Nature Parks. The visitor site is located in the ‘Penguin Parade’, and penguins in this area breed in nest boxes, are checked and handled once a month by trained volunteer researchers and are exposed to nightly tourist activity associated with the Penguin Parade. The order of visits to each site was rotated throughout the study period. The penguins in the research and research/visitor groups all wore flipper bands indicating that they had been captured and handled at least once prior to this study.

### Capture protocol and blood sampling

Each penguin was captured only once. All individuals were captured and handled according to the following procedure, which was adapted from the Capture Stress Protocol, a widely accepted technique for evaluating the sensitivity of the hypothalamic–pituitary–adrenal axis in birds (Wingfield *et al*., 1995). A timer was started immediately before reaching into a penguin burrow. If a penguin was present, it was removed from the burrow, its body wrapped in a towel with its feet and head exposed, and 0.2 ml of blood extracted from an interdigital vein using a 1 ml Tuberculin^©^ syringe with a 25-gauge needle (Becton and Dickinson, Sydney, NSW, Australia). The time at which the first blood sample was obtained was recorded and was always <4 min after capture. Three more blood samples were obtained at prescribed intervals of 10, 20 and 30 min after initial capture, with penguins placed in an open-lid plastic tub between sampling times. Although it was impossible to obtain blood samples at precisely the same time for every individual, all attempts were made to standardize sampling times and to minimize the variation across individuals and study groups. Blood samples were transferred to micro-centrifuge tubes immediately after extraction and kept cool on ice, at ∼4°C, until brought back to the laboratory for centrifugation. All blood samples were centrifuged within 6 h of collection and plasma fractions stored at less than −20°C until radioimmunoassay, which occurred within 4 months of collection.

The penguins were weighed in a calico bag to the nearest 20 g using a 2 kg Pesola^©^ spring balance and sex was determined according to bill morphometrics [bill width, bill depth, culmen length and gonys depth measured to ±0.1 mm using dial callipers (Swiss Precision Instruments)] after the final blood sample had been obtained. A *post hoc* discriminant function analysis was used to determine the accuracy of sex classification (Turner, 2001; for comparable method, see Arnould *et al*., 2004).

### Corticosterone assay

The corticosterone assay followed the radioimmunoassay technique of Wingfield and Farner (1975), but without chromatography and as modified by Ball and Wingfield (1987). Briefly, all plasma samples (ranging in volume from 5 to 20 μl) were diluted with phosphate-buffered saline (to 100 μl total) and equilibrated with 2000 counts per minute (cpm) of tritiated corticosterone (New England Nuclear) at 4°C overnight. Twenty microlitres of tritiated corticosterone (Australia) and 5 ml of scintillation fluid (Omnifluor) were added in triplicate to scintillation vials for total counts and stored in a dark place until counting. Samples were extracted in 5 ml of freshly redistilled dichloromethane. The dichloromethane fraction was aspirated and dried under a stream of nitrogen in a 37°C water bath. The dried extracts were reconstituted overnight in 550 μl of phosphate-buffered saline. A 100 μl aliquot was added to a scintillation vial with 5 ml of Omnifluor and counted directly in a scintillation counter (Wallac Ltd) to determine the percentage recovery. Aliquots of 200 μl were added to duplicate assay tubes. A serial 50% dilution of stock standards (2000 pg/100 μl; Sigma) provided nine standards. The 200 μl aliquots (samples) and the nine standards were incubated with 100 μl of antibody to corticosterone (B3-163; Endocrine Science Labs, Calabasas, CA, USA) and 100 μl of tritiated corticosterone (∼10 000 cpm) overnight at 4°C. The samples and standards were mixed with 0.5 ml of dextran-coated charcoal and allowed to incubate for 12 min at 4°C to separate the bound and free corticosterone. All tubes were immediately centrifuged at 2000 rpm at 4°C for 10 min. Supernatants (containing bound corticosterone) were decanted into scintillation vials, mixed with 5 ml of Omnifluor, and equilibrated for at least 3 h before counting.

All sample results were adjusted according to their respective recovery values. The percentage recoveries for the samples ranged between 73.6 and 86.4%, with a mean of 80.0% for all assays. The variation between duplicate samples was <8.1%. Within each assay, two solvent blanks and two standard samples (one each at the beginning and one at the end) were taken through the entire assay procedure as a way to evaluate the intra- and inter-assay variability. The intra-assay coefficient of variation (CV) was <10.1% and inter-assay CV was 13.2%. The lowest detectable concentration was 0.078 ng/ml.

### Behaviour scoring

Each individual’s behavioural response to handling was evaluated immediately before each bleed. Behaviour was rated on a scale from 0 to 3, as follows: 0 described very submissive or passive behaviour, with minimal struggling and no attempts to escape; 1 described less passive behaviour, with bouts of struggling, wriggling and attempts to escape; 2 described more active behaviour, with frequent struggling, occasional pecking and attempts to escape; and 3 described extremely active and aggressive behaviour toward the handler, which included incessant struggling and pecking, with continuing attempts to escape.

### Statistics

All analyses were performed in R, version 13.4.0 (R Core Development Team, 2015). For corticosterone analyses, we chose to use linear mixed models (LMMs) (function ‘lmer’ in package lme4; Bates *et al*., 2014) rather than repeated-measures ANOVAs because LMMs have less stringent assumptions regarding the variance–covariance matrix, can better accommodate missing observations and have greater flexibility for grouping designs (Magezi, 2015). To determine the influence of sex and time of day on the corticosterone stress response of naïve birds during 30 min of capture, we built LMMs with sample time (4, 10, 20 and 30 min) and all combinations of sex, day/night and their interactions as fixed effects, and individual as a random effect. We assumed the ‘null’ model to include only the random effect (individual) and sample time, because this model represented individual variability in corticosterone concentration at each sample time. Candidate models were compared using Akaike’s information criterion with correction for small sample bias (AICc). To assess the effect of prior exposure to human activity on the shape of the corticosterone stress response, we developed a single LMM with an interaction between site (control, research or research/visitor) and sample time as fixed effects, and individual as a random effect. Both corticosterone analyses used LMMs fitted with maximum likelihood parameter estimation, and a Gaussian distribution and identity link. QQ plots of the residuals of these models suggested that assumptions of normality were reasonable.

For the behaviour analyses, we described the behavioural response at the initial and final sample times (4 and 30 min) during capture. We used ordinal logistic regressions (function polr in MASS; Venables and Ripley, 2002) because the response was multinomial (0–3), and the scale of the penguins’ response was ordered from least to most aggressive. The results of these analyses are reported as proportional odds ratios, which give the probability of the response moving from all lower levels to a higher level, based on a one unit increase in the explanatory variables. To determine which variables caused naïve penguins to be more aggressive, we developed models with corticosterone concentration, sex and day/night as predictors and chose the best model using AICc. To assess the influence of human activity on behaviour and to determine whether the relationship between behaviour and corticosterone differed given a history of exposure to human activity, we used site and corticosterone as predictors.

## Results

### Control site

#### Effects of capture, sex and time of day on corticosterone concentration

At the control site, adequate plasma volumes were collected during at least one sample time from 13 females and 18 males at night, and from a different set of 12 females and 19 males during the day (*n* = 62). Mean corticosterone concentrations were 11.22 ± 3.81 ng/ml at 4 min and 155.59 ± 10.39 ng/ml at 30 min post-capture.

**Figure 1:**
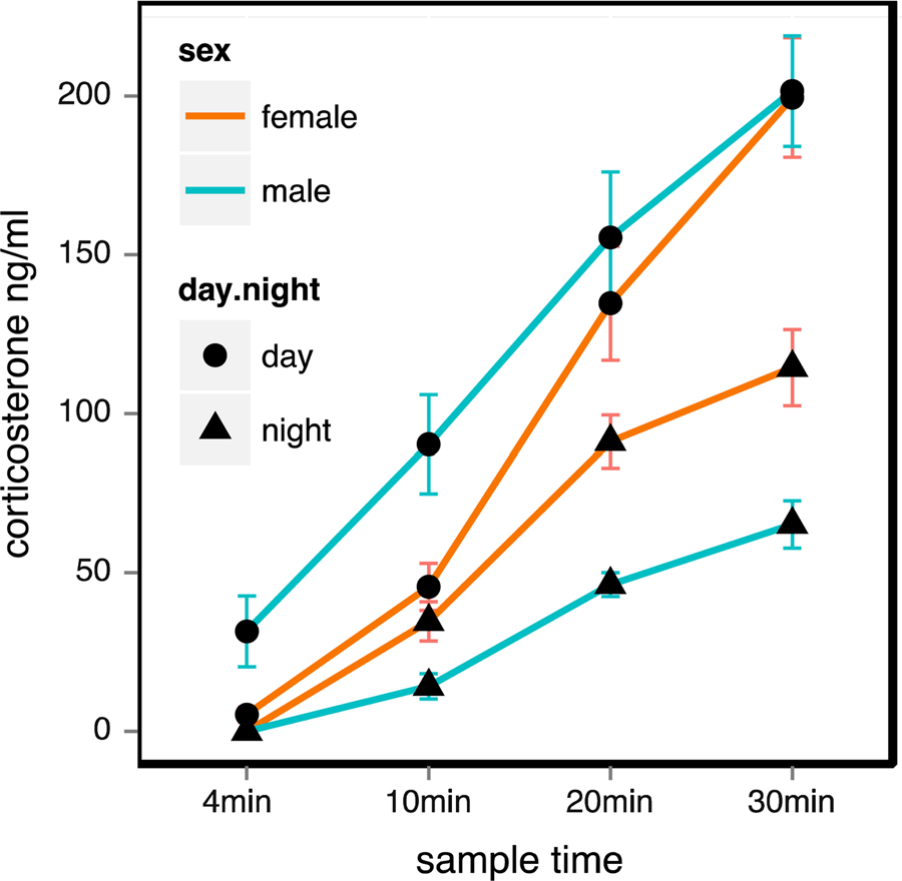
The corticosterone stress response of little penguins that had not previously encountered human activity, throughout 30 min of capture and handling. Penguins are grouped here by their sex and whether measurements were taken during the day or night.

We found that of the nine LMMs that we developed, only three models had noticeable empirical support, with a combined Akaike weight of >0.99 (see Table 1). These models performed substantially better than the null model (>1 000 000 times), indicating that the corticosterone response was not simply a function of individual variability at a given sample time. The three best models all included day/night and sample time and an interaction between these terms. The importance of this interaction term in the models reflects the increase in corticosterone concentration from the baseline at each subsequent sample time (10, 20 and 30 min post-capture) and that the magnitude of this response varied according to the time of day, with penguins having a stronger stress response during the day than at night (Fig. 1). The LMM that included only these effects performed best (Table 2). Although this model had only 2.4 times more evidence than the model that included sex as an additional term, and these models were ranked competitively (<2 ΔAICc; Burnham and Anderson, 2002), it is likely that the sex parameter in the second model was uninformative, because the model fit was not improved enough to overcome the penalty of adding a single additional term (for details, see Arnold, 2010). The third best model included a three-way interaction term between sex, day/night and sample time. This model received 13.4 times less support than the best model. Although visual inspection of the plot suggested an interaction between sex and time of day, with females having a greater stress response than males at night, and males having a greater stress response than females during the day, there was little evidence that the interaction term was informative. As we were primarily interested in relationships between the variables and their effect on corticosterone secretion rather than in the accuracy of predictions or specific parameter estimates, we did not perform model averaging on the three best models, and report estimates solely for the best model.

**Table 1:** Comparison of the top four models evaluating the influence of parameters sex, day vs. night and sample time (4, 10, 20 and 30 min post-capture) on corticosterone concentration of naïve penguins, with ID as a random effect

Model	K	LL	AICc	ΔAICc	W*_i_*
Cort ∼ day vs. night × time + (1 | ID)	10	−920.535	1862.4	0	0.6652
Cort ∼ day vs. night × time + sex + (1 | ID)^a^	11	−920.249	1864.1	1.70	0.2843
Cort ∼ day vs. night × sex × time + (1 | ID)	18	−913.619	1867.5	5.15	0.0506
Cort ∼ day vs. night + time + (1 | ID)	7	−937.982	1890.6	28.23	0.0000
Cort ∼ time + (1 | ID)	6	−951.388	1915.3	52.88	0.0000

The null model (sample time and random effect) is included for comparison. Abbreviations: AICc is Akaike’s information criterion corrected for small sample sizes; ΔAICc shows how well each of the models performs in relationship to the best model; K is the number of model parameters; LL is the log likelihood; and W*_i_* is the Akaike weight of each model, showing the probablility that it is the best model in the set.

a The second model in the set is likely to be uninformative because it adds a single additional parameter and the ΔAICc is within 2 points (Arnold, 2010).

**Table 2:** Maximum likelihood estimates of parameters in the best model describing the corticosterone response in the control group, with standard error and 95% confidence intervals

Fixed effect	Parameter estimates	Standard error	95% Confidence interval
Intercept	15.42	10.22	−4.80, 35.55
Night (day)	−19.48	15.02	−49.12, 10.15
10 min (4 min)	58.62	10.20	38.44, 78.75*
20 min (4 min)	127.36	10.02	107.57, 147.15*
30 min (4 min)	185.81	9.83	166.34, 205.18*
10 min : night	−29.47	15.43	−59.96, 0.94
20 min : night	−55.65	15.22	−85.72, −25.63*
30 min : night	−93.14	15.62	−123.94, −62.29*

Base levels against which other levels are compared in parentheses.

* Effects with confidence intervals not overlapping zero were significant at *P* < 0.05.

#### Effect of capture, corticosterone, sex and time of day on behaviour scores

At the control site, we measured behaviour scores for 10 females at night, 13 males at night, 8 females during the day and 14 males during the day. We compared ordinal logistic regression models for 4 and 30 min behaviour scores, with combinations of corticosterone concentration, sex and day/night as predictors. We found that for both the 4 and 30 min behaviour scores, the models with corticosterone alone performed best based on AICc. However, in this model the effect of corticosterone on behaviour score was minimal at both 4 and 30 min, with proportional odds ratios showing that an increase in corticosterone of 1 ng/ml resulted in penguins being 0.98 [95% confidence interval (CI) = 0.92–1.01] and 1.00 (95% CI = 0.99–1.01) times more likely to be in a more aggressive category, respectively.

### Control vs. impact sites

#### Effects of capture and previous exposure to human activity on corticosterone concentration

Adequate plasma volumes were obtained at night during at least one sample time from 13 females in the control group, 13 in the research group and 13 in the research/visitor group. Data from these 39 penguins were used in the corticosterone analysis. At 4 min, mean corticosterone concentrations were undetectable (<0.078 ng/ml) in the control group, 13.77 ± 12.66 ng/ml in the research group and undetectable (<0.078 ng/ml) in the research/visitor group. At 30 min, concentrations were 114.48 ± 12.02 ng/ml in the control group, 168.68 ± 13.6 ng/ml in the research group and 160.46 ± 17.70 ng/ml in the visitor group.

**Figure 2:**
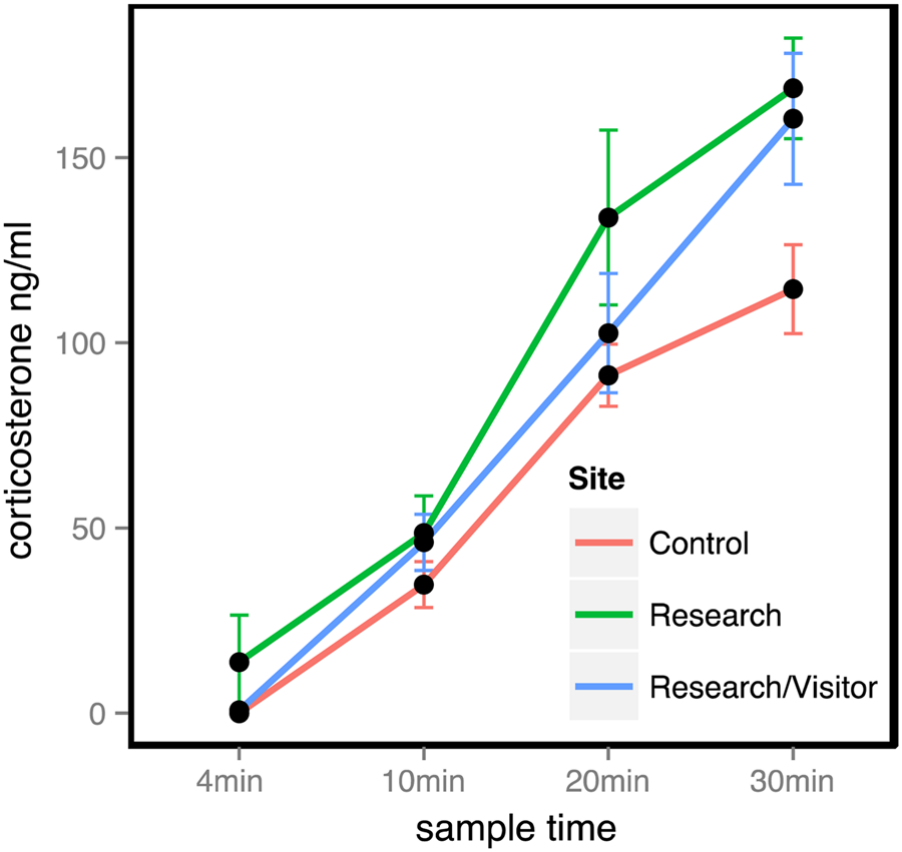
The corticosterone stress response of little penguins at the following three sites: the control site, where penguins had not encountered humans before; the research site, where penguins were handled twice a month for monitoring purposes; and the research/visitor site, where penguins were handled monthly for monitoring and also observed nightly by tourists at the ‘Penguin Parade’.

To understand the effect of prior exposure to human activity on capture stress, we used an LMM with an interaction term between site and sample time (Table 3). Corticosterone concentrations increased at each subsequent sample time in all study groups, although the magnitude and shape of the increase was different between the sites (Fig. 2). The corticosterone stress response was lower in the control group than in either of the impact sites at the 10, 20 and 30 min sampling times. Statistically significant differences by site were observed at 30 min, with concentrations appearing to level off in penguins at the control site relative to both the research and research/visitor sites. Although the mean corticosterone concentration at 4 min was almost 14 times higher in the research group than the control group, this difference was not statistically significant (Welch two-sample *t*-test: d.f. = 10, *t* = −1, *P* = 0.34).

**Table 3:** Maximum likelihood estimates of parameters in the model describing the corticosterone response in relationship to site, with standard error and 95% confidence intervals

Fixed effect	Parameter estimate	Standard error	95% Confidence interval
Intercept	4.23	14.55	−24.53, 33.08
10 min (4 min)	31.77	14.03	4.02, 59.59*
20 min (4 min)	86.77	13.89	59.31, 114.32*
30 min (4 min)	109.77	14.75	80.62, 139.01*
Site R (C)	8.84	19.67	−30.19, 47.78
Site RV (C)	5.26	19.51	−33.34, 43.97
10 min: site R	18.09	18.98	−19.74, 55.56
20 min : site R	32.75	18.61	−4.14, 69.55
30 min : site R	41.46	19.19	3.47, 79.42*
10 min : site RV	6.19	18.84	−31.08, 43.54
20 min : site RV	6.98	18.62	−29.83, 43.89
30 min : site RV	41.08	19.15	3.22, 79.03*

Reference levels are in parentheses. Abbreviations: C, control site; R, research site; and RV, research/visitor site.

* Effects with confidence intervals not overlapping zero were significant at *P* < 0.05.

#### Effect of capture and human activity on behaviour score

**Figure 3:**
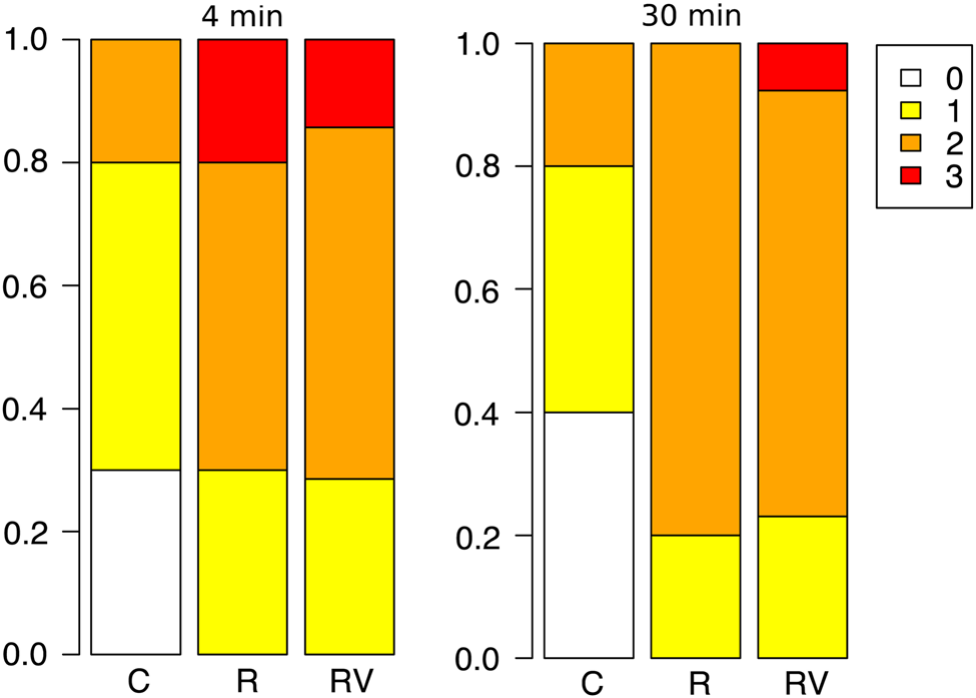
Observations of little penguin behaviour states at three sites (control, research and research/visitor) as a proportional stacked barplot (percentage of penguins in each behaviour state by site); 0 (white) represented penguins in the least aggressive behaviour state, whereas 3 (red) represented penguins in the most aggressive behaviour state.

Behaviour scores were obtained at 4 and 30 min from 10 females at night from the control site, 10 females at night from the research site and 14 females at night from the research/visitor site (Fig. 3). We found a strong relationship between site and behaviour that was significantly improved when corticosterone was included in the model (ΔAICc between 4 min model with and without corticosterone = 17.73; 30 min model with and without corticosterone = 7.87). Proportional odds ratios derived from ordinal logistic regressions showed that at 4 min (given that all other parameters in the model were held constant), being at the research site made a penguin 19.33 (95% CI = 1.81–535.00) times more likely to have a more aggressive behaviour score than those at the control site, being at the research/visitor site made penguins 20.99 (95% CI = 2.11–558.11) times more likely to be more aggressive than those at the control site, while a 1 ng/ml increase in corticosterone made a penguin 0.69 (95% CI = 0.37, 1.15) times more likely to be more aggressive. At 30 min, penguins at the research site were 27.76 (95% CI = 2.81–705.51) times more likely to be more aggressive, and penguins at the research/visitor site were 43.13 (95% CI = 3.79–1228.95) times more likely to be more aggressive than those at the control site, whereas a 1 ng/ml increase in corticosterone made a penguin 1.01 (95% CI = 0.99–1.03) times more likely to be more aggressive.

## Discussion

Physiological mechanisms can signal how effectively animals respond to challenges in their environment before changes in demographic parameters are apparent (Adams and Ham, 2011). In the present study, we found a clear and observable effect of prior exposure to human activity on the acute glucocorticoid stress response of adult female little penguins compared with that of a control group. Although increased stress may be negatively correlated with survival and reproduction in some cases, interpreting the relationship between physiological parameters and biological outcomes can be challenging (Busch and Hayward, 2009). We explore our findings below and discuss how they impact our understanding of penguin ecophysiology, management and conservation.

### The natural stress response

This is the first study of the hormonal stress response in little penguins, and it appears that they have an unusually acute glucocorticoid response to capture relative to other avian species. The initial and 30 min concentrations of corticosterone observed in naïve little penguins are two to nine times greater than those measured in other, larger penguins [gentoo (*Pygoscelis papua*) and king (*Aptenodytes patagonicus*) penguins, Holberton *et al*., 1996; Magellanic penguins (*Spheniscus magellanicus*), Fowler *et al*., 1995; Hood *et al*., 1998; Walker *et al*., 2006; Adélie penguins (*Pygoscelis adeliae*), McQueen *et al*., 1999; Vleck *et al*., 2000; Cockrem *et al*., 2009; Macaroni penguins (*Eudyptes chrysolophus*), Crossin *et al*., 2012]. Most of these penguin studies were conducted during the breeding season, a period in the life cycle when stress levels are commonly elevated in birds (reviewed by Romero, 2002) but may be modulated to avoid nest desertion in species (including some penguins) that breed in harsh conditions and that experience a high cost of nest abandonment (Groscolas *et al*., 2008). Comparisons with other non-breeding birds [e.g. the black-throated sparrow (*Amphispiza bilineata*), Wingfield *et al*., 1992; the white-crowned sparrow (*Zonotrichia leucophrys*), Wingfield *et al*., 1982; Wingfield, 1988; and the American redstart (*Setophaga ruticilla*), Angelier *et al*., 2009] show that concentrations of corticosterone secretion by little penguins during our study may be atypically high for non-breeding birds.

The heightened stress response observed in this study might be a result of the sampling period, which followed the end of the breeding season, the post-nuptial moult and corresponding fasting period. It is possible that little penguins have reduced feedback for a short period following the moulting fast. This would allow them to increase levels of corticosterone secretion, which may help to support a high level of foraging activity (Astheimer *et al*., 1992) and a subsequent rapid weight gain after the moult. Another possibility is that naturally high corticosterone concentrations may provide an evolutionary advantage to little penguins because of their higher energetic requirements for foraging (Bethge *et al*., 1997) and smaller size, which result in a greater threat from terrestrial predators (Dann, 1992) relative to other penguins. Sampling little penguins at other life-history stages will illuminate seasonal patterns in the stress response and confirm whether little penguins demonstrate particularly high corticosterone secretion throughout their annual cycle.

As in other species, the stress response in the little penguin varies in magnitude with time of day (Fig. 1). The corticosterone response in little penguins was more acute during the day than during the night, suggesting a diurnal pattern of corticosterone secretion and/or a heightened stress response to being exposed and challenged on land during daylight (Dann, 2013). The day concentrations were higher at each time interval during capture than the night concentrations and increased significantly with time throughout the entire capture period, whereas the night concentrations levelled off after 20 min. In avian species, circulating corticosterone usually peaks during the inactive period and is at its lowest during the period when the bird is most active (Joseph and Meier, 1973; Dufty and Belthoff, 1997; Breuner *et al*., 1999; Romero and Remage-Healey, 2000; Rich and Romero, 2001). This is potentially dictated by increased demand for corticosterone to produce a sufficient physiological and behavioural response to a stressor during periods of inactivity (Romero and Remage-Healey, 2000). Little penguins are diurnal foragers at sea, whereas on land they spend the day resting inside their burrows, with almost all their social and physical activity occurring after dark. The apparent diurnal pattern of corticosterone secretion that we observed in the present study may therefore be a function of activity level rather than an innate cycle related to the penguins’ circadian rhythm, and may therefore change when the penguins are at sea.

Sex differences in baseline and stress-induced corticosterone concentrations are observed in some, but not all avian species (e.g. Edwards *et al*., 2013; Angelier *et al*., 2015). When they occur, these differences can usually be attributed to different roles played by each sex in the breeding cycle, with corticosterone often being attenuated in the sex responsible for incubation or primary parental care (O’Reilly and Wingfield, 2001). In sexually dimorphic species, differences in body condition resulting from differential breeding investment in relationship to initial body mass can also contribute to sex-specific hormonal stress responses (Lormée *et al*., 2003). Our study was conducted outside the breeding season, and we found no significant differences in the corticosterone concentrations of male and female little penguins (Fig. 1 and Table 1). However, even during breeding there is little evidence for sex differences in the corticosterone stress response of other penguin species (Adelie penguins, Vleck *et al*., 2000; Magellanic penguins, Fowler, 1999). This may be because of the bi-parental care that penguins undertake to raise offspring (Garcia *et al*., 1996) and the approximate similarity in body size between sexes (e.g. Bertellotti *et al*., 2002; Poisbleau *et al*., 2010).

Behaviour can be hormonally mediated, and in some species there are relationships between aggression and corticosterone concentrations, both positive (Van Duyse *et al*., 2004) and negative (Wingfield and Silverin, 1986; Tokarz, 1987). Although numerous captive studies point to corticosterone responses underlying aggressive behaviour expressed in reactive/proactive personalities (reviewed by Cockrem, 2013), others have found weak [Nazca boobies (*Sula granti*), Grace and Anderson, 2014] or no [male collared flycatchers (*Ficedula albicollis*), Garamszegi *et al*., 2012] relationship between the stress response and aggressive behaviour in undisturbed wild birds. We found very slight evidence for a negative relationship between corticosterone and behaviour score at 4 min, and no evidence for either a positive or a negative relationship between corticosterone and behaviour at 30 min. This suggests that in naïve non-breeding penguins, aggressive behaviour appears to be uncoupled from the hormonal stress response (Logan and Wingfield, 1995) and supports the emerging trend that these relationships tend to be weak in wild birds and are likely to depend on testing context (Grace and Anderson, 2014).

### The stress response in relationship to capture vs. capture and visitation by humans

In this study, all penguins exposed to prior capture and handling demonstrated elevated corticosterone concentrations after 30 min of capture compared with the naïve control group (Fig. 2). Overall, there were no clear differences in the stress response at the research (capture) and research/visitor (capture plus controlled visitation/observation by humans) sites, although both groups showed elevated corticosterone concentrations and heightened aggression compared with penguins at the control site. Although we might expect an additive effect of observation by tourists on stress levels in penguins, in this case the relatively benign effect of well-regulated tourism is probably masked by the more traumatic effect of capture during the course of research and monitoring procedures. Future studies could separate the effect of relatively non-invasive visitation by tourists from the more invasive effect of capture and handling by assessing stress hormones in penguins that are exposed to visitors but not routinely handled (e.g. Walker *et al*., 2005, 2006). Ideally, this could be done non-invasively by examining corticosterone concentrations in faeces (e.g. Hämäläinen et al., 2014; Ozella *et al*., 2015). This will help to ascertain whether there is a level of interaction between humans and little penguins that can be maintained without eliciting a physiological effect.

The fact that the penguins in the present study did not habituate to handling is perhaps surprising, because other species appear to become habituated to human activity. For example, Magellanic penguins exposed to frequent visitation but no routine capture show lower corticosterone concentrations after being exposed to a simulated tourist visit for 15 min than penguins in a non-tourist area (Villanueva *et al*., 2012) and lower concentrations after 30 min of capture compared with penguins in a control area, despite showing no difference in baseline concentrations in either study (Walker *et al*., 2006; Villanueva *et al*., 2012). The same pattern of similar baseline but lower concentrations of corticosterone secretion during capture was observed in Galapagos iguanas (*Amblyrhynchus cristatus*) exposed to visitation by tourists (Romero and Wikelski, 2002). Yellow-eyed penguins (*Megadyptes antipodes*) exposed to unregulated tourism show similar patterns of corticosterone secretion during capture to the penguins in our study, with a heightened response in disturbed individuals after 15 min of capture (Ellenberg *et al*., 2007). Although even repeated high-intensity interactions, such as handling, can lead to habituation in other species [e.g. American kestrels (*Falco sparverius*), Love *et al*., 2003], it appears that in some species, including little penguins, there may be a type or level of human interaction to which habituation cannot occur.

In this study, the penguins in the research and research/visitor groups also exhibited more aggressive behaviour than the control group. At both 4 and 30 min post-capture, they had a greater tendency to struggle, peck and attempt to escape than penguins at the control site. This is a further indication that the research and research/visitor penguins have not habituated to capture and handling but rather resist it with high-energy aggressive behaviour. Corticosterone also seemed to have a stronger effect on behaviour scores in the analysis that included prior human interaction. There was a negative relationship between corticosterone and behaviour at 4 min, indicating that once the effect of site was accounted for, some penguins were initially aggressive even when they had low baseline corticosterone concentrations. This could perhaps be explained if the level of aggression is enhanced (or learned) through repeated capture and is initiated before stress hormone concentrations rise. Conversely, at 30 min, there was a positive relationship between corticosterone concentration and behaviour score when site was taken into account. This suggests that at the impact sites, corticosterone may function to allow penguins to sustain a heightened aggressive response to repeated capture, by mobilizing energy stores (Sapolsky *et al*., 2000).

The lack of a significant difference in corticosterone concentrations at all study sites at 4 min and the significant differences between the control and both impact sites at 30 min suggest that the effects of human activity are generally unseen in baseline concentrations but are likely to be expressed in the form of enhanced adrenal potential or reduced feedback (Astheimer *et al*., 1992; Walker, 1994). This pattern is generally seen in animals that frequently secrete high concentrations of corticosterone in response to unpleasant stimuli and therefore develop mechanisms to enhance and prolong the stress response. The similar baseline concentrations across all three sites indicates no evidence for chronic stress caused by research and tourism in this species. The significant elevations in corticosterone during capture in all groups also indicates that penguins affected by disturbance are able to respond effectively to unexpected events and do not exhibit desensitization of the hypothalamic–pituitary–adrenal axis, a sign of chronic stress (Rich and Romero, 2005). These are positive findings for the conservation and management of little penguins, as it appears that the intensity of human interactions with penguins occurring at the time of the study (i.e. capture and handling twice per month, or capture and handling once per month plus observation by tourists) was not sufficiently high to elicit chronic stress; hence it would not be likely to contribute to a serious allostatic overload.

Although sensitization of the stress response was apparent in the present study, care must be taken not to automatically see any increase in stress as being inherently negative, as an appropriate response to challenges will be positively related to survival if it enables an animal to respond effectively to disturbances such as predation or intraspecific aggression. However, although an enhanced adrenal response is likely to confer benefits to individuals when faced with immediate threats to their survival, repeated exposure to capture by humans does not pose such a threat, and the energy routinely spent on aggressive behaviour is ‘wasted’. The increase in energy expenditure mobilized by heightened corticosterone and used for aggressive interactions with humans is a particularly important consideration during the breeding season, when energetic demands are highest (Gales and Green, 1990). Heightened corticosterone concentrations also appear to mediate egg and chick abandonment in other penguins, and this may be of particular concern for energy-depleted little penguins, particularly those brooding their second clutch (Groscolas *et al*., 2008; Angelier and Chastel, 2009).

Small alterations to research and tourism protocols may make a difference to the stress experienced by animals. In the case of little penguins, areas of a colony exposed to the greatest disturbance by visitors may benefit from being excluded from the monitoring programme or experimental protocols, thereby allowing them to habituate to the more benign presence of tourists without the stress of capture and handling (Walker *et al*., 2006). Based on the results of this study, any handling necessary for research or monitoring may be best performed in the late afternoon or at night during the period when corticosterone secretion is lowest. In our study area, handling related to research has been reduced significantly since the sampling period in 2000 through the introduction of automatic individual identification and weighing systems. The effect of introducing such non-invasive monitoring techniques, compared with alternative handling regimens, should be studied in this and other species to quantify the benefits of using non-invasive monitoring techniques to study wild animals.

In any monitoring programme, the tangible benefits of interacting with animals to improve conservation outcomes must be weighed carefully against the potential cost to individual fitness caused by disturbance. Clear aims and defined goals of monitoring programmes are essential to justify their implementation. As our study and others (e.g. Ellenberg *et al*., 2007) show, it should never be assumed that the capture and handling of animals is not stressful or significant for the individuals involved. Studies should always be conducted to understand the impacts of disturbance, preferably using both physiological and fitness metrics, so that we can refine protocols if necessary and avoid contributing to the decline of sensitive populations.

## Funding

This work was supported by Macquarie University. G.C. receives a Macquarie University Research Excellence Scholarship.
